# In vivo pharmacokinetic, pharmacodynamic and brain concentration comparison of fentanyl and *para-*fluorofentanyl in rats

**DOI:** 10.1007/s00204-024-03887-z

**Published:** 2024-10-17

**Authors:** Jeremy R. Canfield, Jon E. Sprague

**Affiliations:** Bowling Green State University, The Ohio Attorney General’s Center for the Future of Forensic Science, 116 Life Science Building, Bowling Green, OH 43403 USA

**Keywords:** Fentanyl, *Para*-fluorofentanyl, Rat, Pharmacokinetics, Pharmacodynamics, Brain region concentrations

## Abstract

In 2022, *para-*fluorofentanyl (pFF) rose to the 6th most reported drug and the most reported fentanyl analog in the United States according to the Drug Enforcement Administration (DEA). pFF differs from fentanyl by the addition of a single fluorine group. To date, pFF has not been extensively evaluated in vivo and is frequently seen in combination with fentanyl. In the present study, the pharmacodynamic (PD) and pharmacokinetic (PK) properties and brain region-specific concentrations of pFF were evaluated in male Sprague–Dawley rats and compared to fentanyl. A 300 μg/kg subcutaneous dose of fentanyl or pFF was administered to assess PD and PK parameters as well as brain region concentrations. PD parameters were evaluated via a tail flick test to evaluate analgesia and core body temperature to measure hypothermia, a surrogate marker of overall opioid toxicity. Fentanyl and pFF were found to be equally active at the tested dose in terms of tail flick response with both compounds producing an analgesic response that lasted up to 240 min post-drug treatment. pFF induced a significantly greater hypothermic effect compared to fentanyl with a maximum temperature decrease of −5.6 ℃. Plasma PK parameters (T_1/2_, AUC, etc.) did not differ between fentanyl and pFF. However, pFF concentrations in the medulla, hippocampus, frontal cortex and striatum were more than two times the fentanyl concentrations. The increase in brain concentrations and greater hypothermic effect suggests that pFF is potentially more dangerous than fentanyl.

**Supplementary Information:**

The online version contains supplementary material available at 10.1007/s00204-024-03887-z.

## Introduction

**Fig. 1:**
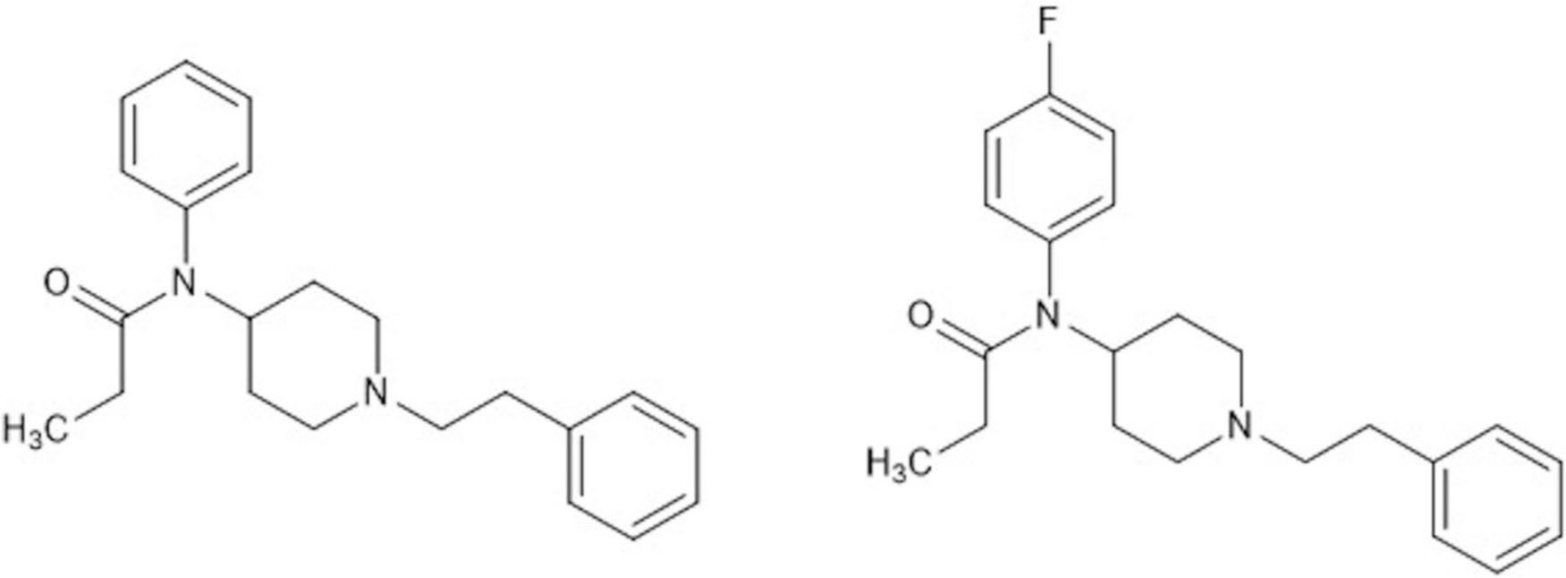
Structures of fentanyl (left) and *para*-fluorofentanyl (right)

*Para*-fluorofentanyl (pFF) is a fentanyl analog that has drastically increased in prevalence across the United States in recent years, and in 2022 was the most frequently seen fentanyl analog according to the Drug Enforcement Administration (DEA) (Diversion Control Division, U.S. DEA, 2023). pFF differs from fentanyl by the addition of a fluorine group to the aniline ring structure of fentanyl (See Fig. 1). The National Forensic Laboratory Information System (NFLIS) reported 7,199 cases of pFF in 2021 which increased to 22,220 cases in 2022 (12,793 of these cases were reported as “fluorofentanyl (unspecified isomer).” Fluorofentanyl, in general, rose from the 12th most reported drug in the United States in 2021 to the 6th most reported drug in the United States in 2022 with pFF likely comprising a majority of these reported cases (Diversion Control Division, U.S. DEA 2022, Diversion Control Division, U.S. DEA, 2023). pFF, along with fentanyl and other fentanyl analogs (referred to as illicitly manufactured fentanyls (IMF), contributed to an estimated 74,702 of the total 107,543 drug overdose deaths in 2023 or nearly 70% (Centers for Disease Control and Prevention, 2024). IMF, which are Schedule I controlled substances in the United States, are produced clandestinely and have not been evaluated for medicinal purposes. The earlier emergence of IMF on the market was in an attempt to circumvent federal law by changing the chemical structure and making new compounds that were no longer illegal (Armenian et al. 2018; Brunetti et al. 2021; Pergolizzi et al. 2021; Schueler 2017). However, a class-wide ban on fentanyl analogues in 2018 resulted in these compounds being scheduled and there was a general shift to non-fentanyl opioid agonists (e.g., nitazenes) with the noted exception of pFF (Weedn et al. 2021).

Halogenation of a drug has the potential to increase the half-life and lipophilicity of a drug, as well as increase membrane permeability and receptor binding (Gerbtzoff et al., 2004; Gunaydin et al. 2018; Rosa et al. 2015). Many studies involving IMF are conducted in vitro to attempt to characterize metabolites or mu opioid receptor (MOR) binding and activation potential. Therefore, the effects of halogenation of the fentanyl pharmacophore in humans are unknown due to limited in vivo studies. Of the studies that have been conducted in vitro*,* differing results of potency of the two compounds have been reported. Hassanien et al. (2020) determined fentanyl to have a binding affinity of 1.6 nM and pFF a binding affinity of 4.2 nM to the MOR. Using a GTPγS assay, Hassanien et al. (2020) further found fentanyl to have a lower EC_50_ than pFF with values of 32 nM and 79 nM, respectively. This differs from the EC_50_s determined by Ulens et al. (2000) using electrophysiology-based techniques, where those authors found just the opposite with pFF having a lower EC_50_ than fentanyl with values of 4.2 nM and 28.8 nM, respectively (Hassanien et al. 2020; Ulens et al. 2000). In addition, Kanamori et al. (2021) also found differing EC_50_ values from the aforementioned studies with fentanyl having a lower EC_50_ of 0.35 nM and pFF displaying an EC_50_ of 0.51 nM (Kanamori et al. 2021). Utilizing different analgesic animal models, in vivo studies have found pFF to be equipotent to fentanyl (Varshneya et al. 2023) or that pFF was less potent than fentanyl (Higashikawa and Suzuki 2008). Therefore, due to the conflicting in vitro and in vivo studies, the possibility exists that halogenation of the fentanyl core structure can lead to increased blood brain barrier (BBB) crossing and more potent binding of the MOR resulting in altered pharmacokinetic (PK) and pharmacodynamic (PD) effects (Rosa et al. 2015; Gentry et al. 1999). These two qualities together could make pFF more potent than fentanyl leading to toxic effects and fatal overdoses in unknowing users or those who can be unintentionally exposed to this compound.

Given the aforementioned effects of halogenation on the toxicology of a compound, we hypothesized that pFF would have altered PK and PD effects when compared to fentanyl. In order to test this hypothesis, we evaluated the plasma PK and PD profiles of pFF compared to fentanyl in a rat model. We also examined the brain concentrations of fentanyl and pFF in the hippocampus, medulla, striatum and frontal cortex.

## Materials and methods

### Chemicals and reagents

Cerilliant (Round Rock, TX) standard solutions of fentanyl, *para*-fluorofentanyl (pFF) and fentanyl-D5 were purchased from Sigma-Aldrich in methanol for preparation of calibration curves (St. Louis, MO). Fentanyl-D5 was used as an internal standard for both compounds. Lithium heparin Sprague–Dawley rat plasma that was used as the matrix for plasma samples was obtained from Innovative Research (Novi, MI). Fentanyl hydrochloride and *para-*fluorofentanyl hydrochloride for animal experiments were purchased from Cayman Chemical Company (Ann Arbor, MI). Drug solutions for animal treatments were prepared fresh each day and were made in saline (Moltox, Boone, NC) using hydrochloride salt forms of fentanyl and pFF at a concentration of 300 μg/mL. Mobile phase A consisted of 0.1% acetic acid (100% LC–MS grade) purchased from Millipore Sigma (Darmstadt, Germany) and water (LC–MS grade) purchased from Fisher Chemical (Waltham, MA). Mobile phase B consisted of pure methanol (LC–MS grade) from Fisher Chemical (Waltham, MA). Formic acid (LC–MS grade) and acetonitrile (LC–MS grade) were purchased from Fisher Chemical (Waltham, MA). The compressed 5.0 ultra-high purity (UHP) grade argon gas tank used for the collision gas was purchased from Linde (Danbury, CT). The nitrogen gas tank that was used for the evaporation step was a nitrogen compressed gas tank obtained from Linde (Danbury, CT). A nitrogen generator was used to supply the heating, drying, and nebulizing gases from SouthTek (Wilmington, NC).

### Standard preparation

A stock solution was prepared by combining and then diluting the two individual methanol standards to a concentration of 1,000 ng/mL in methanol. This solution was then further diluted to 100 ng/mL and 10 ng/mL in methanol to create stock solutions for all calibrators. The calibrators, when spiked into plasma (100 μL), were at final concentrations of 100, 75, 50, 25, 10, 5, 1, and 0.5 ng/mL. Quality control methanol stocks were created by combining and then diluting individual standards to a concentration of 800 ng/mL (high QC), 40 ng/mL (medium QC) and 1,500 ng/mL (low QC) in methanol. The low QC stock was then further diluted to a final concentration of 15 ng/mL in methanol. When spiked into plasma (100 μL), the final concentrations of each quality control were as follows: 80 ng/mL (high QC), 40 ng/mL (medium QC), and 1.5 ng/mL (low QC). The internal standard solution was prepared by diluting fentanyl-D5 to a final concentration of 100 ng/mL in methanol. All solutions were stored in the freezer at −20 ℃ in amber vials until use.

### Plasma preparation methods

Plasma was prepared using protein precipitation with chilled crash solution (1% formic acid in acetonitrile). Briefly, 300 μL of crash solution, 100 μL of spiked, blank, or animal experiment plasma and 30 μL of internal standard solution were aliquoted to a 1.5 mL microcentrifuge tube and placed in an Eppendorf F1.5 ThermoMixer (Eppendorf, Enfield, CT) for 10 min at a speed of 1500 rpm. The crashed plasma sample was then centrifuged for 5 min at 7,900 rpm. The supernatant was drawn off, transferred to HybridSPE phospholipid filters (Supelco, Bellefonte, PA) and extracted by vacuum. The remaining acetonitrile in the extractant was evaporated by a direct flow of nitrogen gas. The extractant was then reconstituted in 100 μL of water + 0.1% acetic acid and injected at an injection volume of 3 μL onto the liquid chromatography tandem mass spectrometer (LC–MS/MS).

### Brain region preparation methods

Individual brain regions were weighed and then processed by tissue homogenization. Briefly, 500 μL of mobile phase A was aliquoted to a 1.5 mL microcentrifuge tube. Then, the brain region sample was added and homogenized using a Benchmark D1000 Homogenizer (Benchmark Scientific, Sayreville, NJ) for 10 s on a setting of 2. The homogenized sample was then centrifuged for 5 min at 7,900 rpm. 100 μL of supernatant was drawn off, transferred to a microvial insert and 30 μL of internal standard solution was added. A corresponding calibration curve containing both fentanyl and pFF was prepared in mobile phase A and all samples and calibrators were then injected at an injection volume of 3 μL onto the liquid chromatography tandem mass spectrometer (LC–MS/MS). The method used was modified from our previously validated plasma method for fentanyl (Canfield and Sprague 2023) to include pFF. Mobile phase A was used as the matrix for all calibrators to correspond with the solution used for brain homogenization. Concentrations of both compounds in each brain region were then quantified using LabSolutions Insight software (version 5.93) and then the concentration per tissue was calculated in ng/g. In order to evaluate for any potential matrix effects, five (5) samples of pre-frontal cortex were used from rats that were not treated with fentanyl or pFF. We utilized the post-extraction addition method for assessing matrix effects according to the Academy Standards Board: Standard Practice for Method Validation in Forensic Toxicology (American Academy of Forensic Sciences, 2019). The area under the curve of five post-extraction addition high concentration quality control (HQC) brain samples were compared to the area under the curve of five neat HQC samples. Matrix effects were deemed acceptable within ± 25%.

### Liquid chromatography tandem mass spectrometry methods

A triple-quadrupole LCMS-8050 CL from Shimadzu U.S.A. manufacturing (Canby, OR) was utilized for sample analysis with a gradient separation method. The method utilized water + 0.1 vol% acetic acid and 100% methanol for the mobile phases and the flow rate was a constant 0.75 mL/min at 70 ℃ over the 6.75 min gradient between 1 and 99% MeOH with a total method run time of 10 min. The gradient began with 1% mobile phase B until 5 min and then the concentration increased to 55% mobile phase B. The gradient then held until 6.76 min where the concentration again increased to 99% mobile phase B. This was held until 8 min where equilibration occurred before the next sample by decreasing mobile phase B back to 1% until the end of the 10-min run. The stationary phase consisted of a Raptor 50 mm X 2.1 mm, 2.7 µm, biphenyl column (Restek, Bellefonte, PA) for the separation of the analytes and a Raptor guard column (Restek, Bellefonte, PA). MRM transitions, Q1 and Q3 pre-bias voltages, collision energy, and retention time for each compound are displayed in Table S1. Concentrations were quantified using LabSolutions Insight software (version 5.93). Representative chromatograms for a plasma calibrator and plasma sample for fentanyl and pFF are displayed in Figure S1. Representative chromatograms for a brain region calibrator and brain region sample for fentanyl and pFF are displayed in Figure S2.

### Method validation

The method used for drug quantification in plasma was previously validated for fentanyl (Canfield and Sprague 2023) according to the Academy Standards Board: Standard Practice for Method Validation in Forensic Toxicology (American Academy of Forensic Sciences, 2019). Therefore, the plasma methods for pFF only were validated prior to sample testing. Parameters assessed were calibration model, bias, precision, limit of detection (LOD), lower limit of quantification (LLOQ), ionization suppression/enhancement, carryover and interference studies.

Calibration models/linearity were determined using the 8 non-zero calibrators indicated above over 5 days in rat plasma matrix samples. Models were then evaluated using residual plots and R^2^ values.

LLOQ and LOD were both defined as the lowest non-zero calibrator (0.5 ng/mL) and were assessed in triplicate over 3 days. Precision (within-and between-run) and bias were assessed for LLOQ and LOD, and % CV and % bias were calculated and determined to be acceptable within ± 20%. Precision (within- and between-run) and bias were assessed using three QC (quality control) concentrations (high, medium, and low) in triplicate over 5 runs in rat plasma. % CV and % bias were then calculated and determined to be acceptable within ± 20%. % CV values for within-run and between-run precision for all LLOQ, LOD and QC samples were calculated using the ANOVA approach as defined in the Academy Standards Board: Standards Practice for Method Validation in Forensic Toxicology Sect. 8.2.2.3.4 (American Academy of Forensic Sciences, 2019).

Ionization suppression/enhancement was determined using the post-extraction addition (American Academy of Forensic Sciences, 2019). Ten different lots of Sprague–Dawley rat plasma were fortified with low and high QCs and ISTD before and after extraction. Ten neat samples were also prepared at the low and high QC concentrations in mobile phase A. Matrix effects were determined by comparing area ratios of post-extraction samples to neat samples and extraction recovery was also determined by comparing the area ratios of pre- and post-extraction samples. Matrix effects were deemed acceptable within ± 25%.

Carryover was assessed by reinjection of an extracted blank matrix after the highest calibrator over 5 days. Carryover was determined to be negligible if analyte response in the blank was < 10% of the analyte response in the lowest calibrator.

Matrix interferences were assessed by the analysis of ten different lots of Sprague–Dawley rat plasma that were not fortified. This was to ensure the matrix itself was not interfering with pFF. Internal standard interference was assessed by analyzing a blank sample with internal standard to assess whether the internal standard was interfering with the detection of pFF. Analyte interference was assessed by analyzing a spiked sample without the addition of internal standard. Exogenous interferences were not assessed for this method due to the studies involving controlled drug-administration to drug-free rats.

### Animal experiments

For PK/PD experiments, animals were housed one per cage (cage size: 21.0 × 41.9 × 20.3 cm) at a room temperature of 24–25 ℃, maintained on a 12:12-light/dark schedule. Food and water were provided ad libitum. The same was true for the brain concentration study except animals were housed two per cage and were given a 1-week acclimation period. Animal maintenance and research were conducted in accordance with the eighth edition of the Guide for Care and Use of Laboratory Animals as set forth by the National Institutes of Health, and all protocols were approved by the Bowling Green State University Animal Care and Use Committee (Protocol Number: 2109741–2). All methods were carried out in compliance with relevant institutional, Federal and ARRIVE guidelines and regulations. At the completion of the study, animals were euthanized by carbon dioxide exposure.

### Pharmacokinetic/pharmacodynamic experiments

Jugular vein cannula (JVC) male, Sprague–Dawley rats were obtained from Envigo (Indianapolis, IN). Seventeen animals were randomly allocated to one of three treatment groups: fentanyl (*n* = 6; 326.92 ± 3.99 g), *para-*fluorofentanyl (n = 6; 330.00 ± 4.49 g), and a saline control (n = 5; 325.90 ± 4.00 g). Time points for the study were 0 min, 30 min, 60 min, 120 min, 240 min, and 480 min post-dosing with 300 μg/kg sc. dose of each compound or 300 μL/kg saline. This dose was determined by a preliminary righting reflex study performed in our lab and based on our previous study (Canfield and Sprague 2023) and on the maximum dose of cyclopropylfentanyl used by Bergh et al. (2021). For pharmacokinetic evaluation, 400 μL of blood was drawn and then centrifuged at 7,900 rpm for 5 min and the plasma was pipetted into a microcentrifuge tube and placed in the freezer at −20 ℃ until analysis. On the day of LC–MS/MS testing, a fresh calibration curve was made, and the calibrators underwent the same extraction procedure as the samples. Concentrations of each compound were then quantified using LabSolutions Insight the same way as with the validation. Following the blood draw, pharmacodynamic parameters were assessed by performing a tail flick assay and measuring basal core body temperature at each time point. Core body temperature was recorded using a rectal probe thermometer. The rectal probe thermometer was a Thermalert Model TH-8 Temperature Monitor obtained from Physitemp (Clifton, NJ). The probe was lubricated with petroleum jelly and gently inserted into the rectum until a steady temperature was reached. Tail flick was assessed using a tail flick analgesia meter obtained from PanLab (Barcelona, Spain). The tail flick assay was performed on the middle third of the tail in triplicate using a focus of 70 and a 10 s cutoff to prevent tissue damage. After the baseline was established, if the tail flick assay timed out then subsequent replicates were not conducted.

### Brain concentration experiments

To determine the brain concentrations of fentanyl and pFF, 12 male, Sprague–Dawley rats were obtained from Envigo (Indianapolis, IN). The 12 animals were randomly allocated to one of two treatment groups: fentanyl (*n* = 6; 355.00 ± 2.86 g) and *para-*fluorofentanyl (*n* = 6; 356.17 ± 1.25 g). Animals were treated with a 300 μg/kg sc. dose of either fentanyl or pFF. After 1 h, animals were killed by carbon dioxide exposure and the medulla, frontal cortex, hippocampus and striatum were dissected on ice. A blood sample was also obtained at this time point and processed as described above. Brain samples were immediately flash-frozen in liquid nitrogen and then transferred to a -80 ℃ freezer until analysis. Brain region concentrations of both drugs were determined using LabSolutions Insight (version 5.93) in ng/mL and then converted to ng/g of tissue using the weights of the whole tissue. A brain to plasma ratio was then calculated based on brain concentration of fentanyl or pFF in each region divided by the plasma concentration of the corresponding compound taken after 1 h in the same animal.

### Pharmacokinetic and statistical analyses

All parent compound plasma concentration versus time rectangular plots were constructed and noncompartmental analysis was conducted utilizing Phoenix WinNonlin software (Version 8.3) to estimate PK parameters. The plasma C_max_ was determined to be the highest observed plasma concentration and the corresponding timepoint was determined to be the T_max_. The terminal elimination rate constant (λz) was calculated by linear regression of the observed terminal natural log concentration versus time data and half-life (T½) was calculated as 0.693/λz. Pharmacokinetic data are presented as the mean ± S.E.M. for both study groups. The AUC_0-∞_ represents the total area under the plasma curve from time zero to infinity and was calculated using the linear trapezoidal rule with the terminal AUC being calculated as the last measured concentration divided by λz. V_D_/F is the ratio of the total dose present in the body to the plasma concentration when the distribution of the drug between the tissues and the plasma is at equilibrium following extravascular dosing. The Cl_p_/F is the plasma clearance of the drug and is calculated as dose/AUC_0-∞_.

All statistical analyses of data were performed using R (version 4.2.2). Raw time course data for tail flick response and body temperature were normalized to percent maximum possible effect (%MPE, 10 s) or change from baseline for each rat (Δ temperature in °C), respectively. MPE was calculated using the following equation:

$$\frac{{{\text{Experimental measure {-} baseline measure}}}}{{{\text{Maximum possible measure {-} baseline measure }}}}{ } \times { 100}$$

Normalized data are presented as mean ± S.E.M, where applicable. The normalized time course data were analyzed by an ANOVA followed by a Tukey’s post hoc test. To calculate the maximum change in temperature (maximum ∆ ℃), the maximum increase or decrease in core body temperature was compared to the animal’s baseline temperature. Maximum change in temperature was analyzed using a Student’s t test between fentanyl and pFF groups. Plasma concentrations and brain concentrations are presented as mean ± S.E.M, where applicable. All plasma pharmacokinetic parameters and brain concentrations were compared between fentanyl and pFF using a Student’s t test. All differences were considered significant with a *p* value < 0.05.

## Results

### Method validation

Plasma validation parameters for pFF are displayed in Table S2. All parameters were within range and were deemed acceptable. The calibration points were validated in the range of 0.5 ng/mL to 100 ng/mL. Within this range, a linear model was accepted with 1/x^2^ weighting and residual plots displayed a random pattern. All R^2^ values were greater than 0.99 and pFF displayed acceptable bias and precision at the LLOQ, as well as for the low-, medium- and high-quality controls (QCs). Matrix effects were less than 25% and recovery was greater than 87% for both the low and high QCs. Carryover was less than 10% in the blank that was reinjected after the highest calibrator and no interferences were observed. Therefore, this method was acceptable to use for plasma samples. Brain matrix effects were within ± 25% for both compounds indicating no ion suppression or enhancement for brain region quantification of either compound (Table S3).

### Plasma pharmacokinetics

**Fig. 2:**
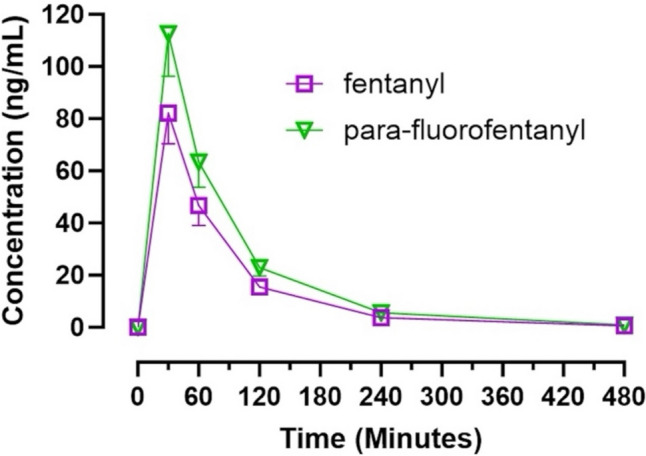
Plasma concentrations versus time plots for fentanyl and *para*-fluorofentanyl. Both compounds were administered at a dose of 300 µg/kg sc. Each value is the mean ± SEM (*n* = 6)

The data in Fig. 2 display the plasma concentration versus time values for fentanyl and pFF. The corresponding PK parameters are displayed in Table 1. Fentanyl displayed a T_1/2_ of 1.10 ± 0.01 h, while pFF displayed a similar T_1/2_ of 1.12 ± 0.03 h. Both compounds also resulted in similar AUC_0-∞_ of 112.83 ± 10.82 ng/mL X h and 158.77 ± 21.60 ng/mL X h for fentanyl and pFF, respectively. For all parameters assessed, there were no significant differences observed between fentanyl and pFF.

**Table 1:** Pharmacokinetic comparison of fentanyl and *para*-fluorofentanyl

	Fentanyl	*para*-fluorofentanyl
t_1/2_ elimination (hr.)	1.10 ± 0.01	1.12 ± 0.03
AUC_0-∞_ (ng/mL X h)	112.83 ± 10.82	158.77 ± 21.60
C_max_ (ng/mL)	82.12 ± 11.78	112.57 ± 16.26
T_max_ (h)	0.5	0.5
Cl_p_/F (mL/h)	917.60 ± 110.11	697.02 ± 109.87
V_D_/F (mL)	1524.90 ± 368.52	1121.11 ± 168.08

Main pharmacokinetic estimates of fentanyl and *para*-fluorofentanyl in the plasma after subcutaneous administration (300 μg/kg) in male Sprague–Dawley rats. Parameters were calculated from plasma concentration versus time plots depicted in Fig. 2 with noncompartmental analysis. Each value is the mean ± S.E.M (*n* = 6)

### Pharmacodynamic effects

**Fig. 3:**
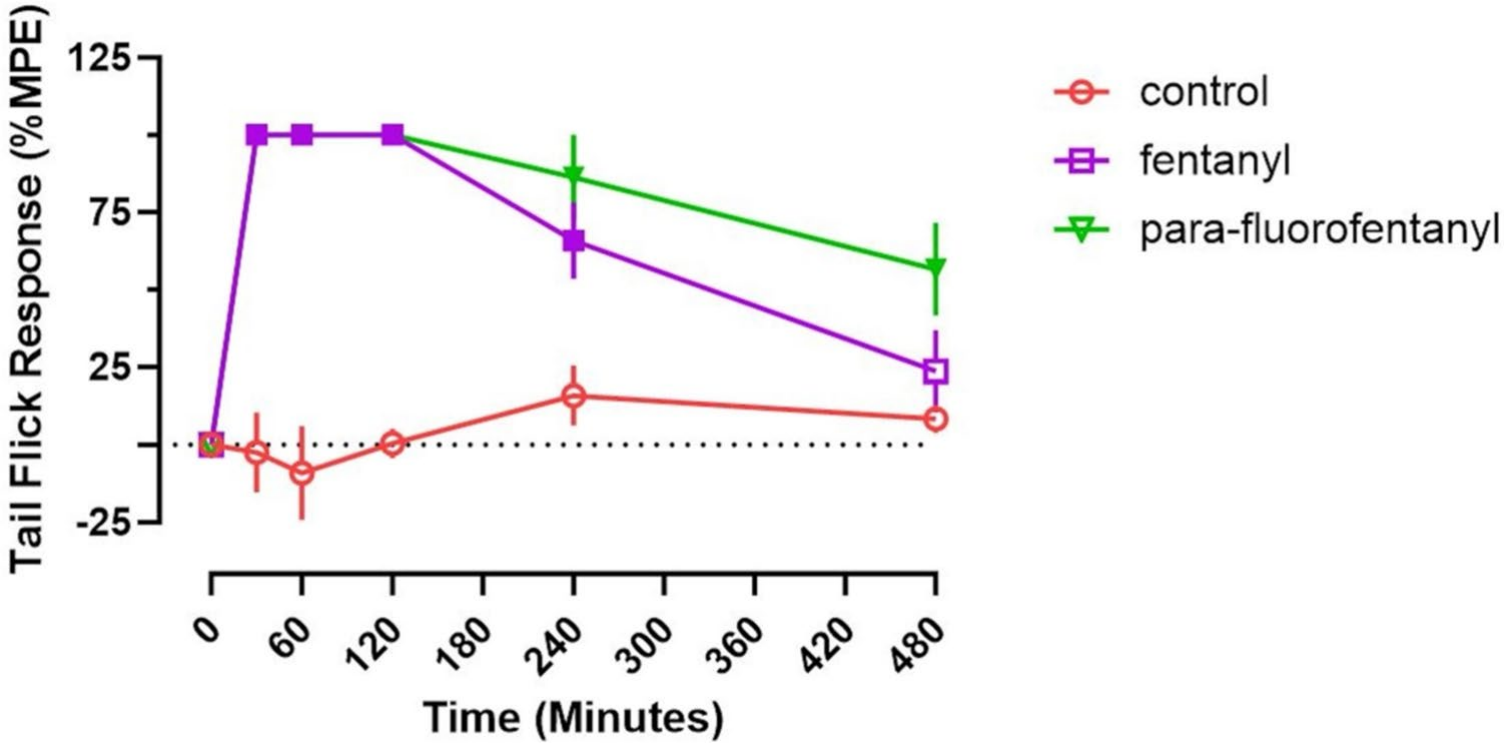
Analgesic effects of fentanyl and *para*-fluorofentanyl assessed by tail flick assay. Compounds were dosed at 300 µg/kg sc. Control animals received saline vehicle (300 µL/kg, sc). Filled symbols indicate significant difference from the control group (*p* < 0.05, one-way ANOVA, Tukey’s HSD post hoc). Each value is the mean ± SEM (*n* = 5–6)

Figure 3 displays the effects of fentanyl and pFF on tail flick response. Fentanyl and pFF were both statistically different from the control group at 30 min (F_(2,14)_ = 78.71, p < 0.001), 60 min (F_(2,14)_ = 63.77, p < 0.001) and 120 min (F_(2,14)_ = 541.6, p < 0.001) indicating an analgesic response compared to the control group by one-way ANOVA with a Tukey’s HSD post hoc. At 240 min, both fentanyl (F_(2,14)_ = 8.12, p = 0.035) and pFF (F_(2,14)_ = 8.12, p = 0.004) displayed significant analgesic effects compared to the control (one-way ANOVA, Tukey’s HSD post hoc). At 480 min, pFF only (F_(2,14)_ = 3.806, p = 0.046, one-way ANOVA, Tukey’s HSD post hoc) displayed analgesia compared to the control but was not significantly different from the fentanyl treatment group (F_(2,14)_ = 3.806, p = 0.174, one-way ANOVA, Tukey’s HSD post hoc).

**Fig. 4:**
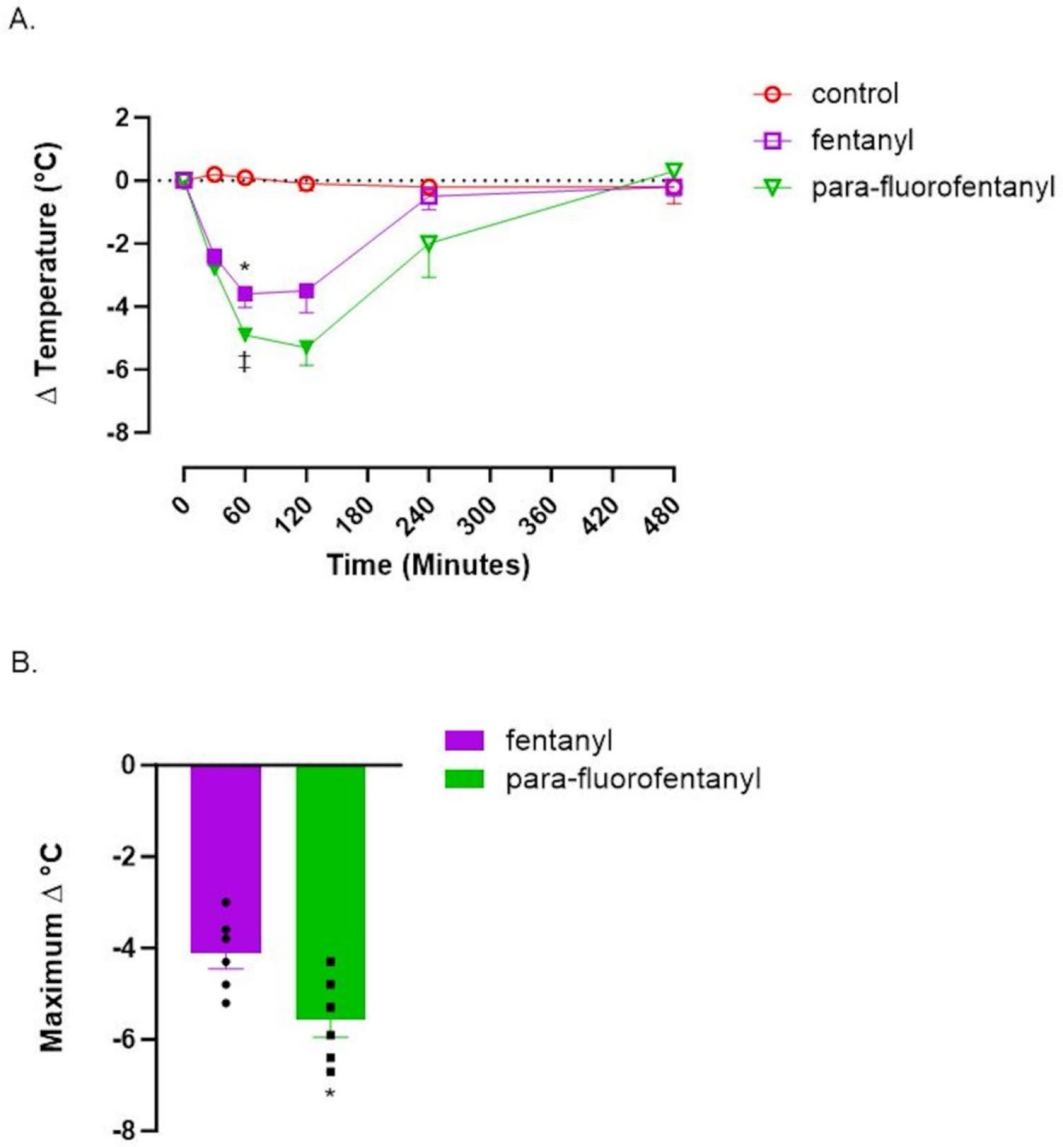
Hypothermic effects of fentanyl and *para*-fluorofentanyl. Compounds were dosed at 300 µg/kg sc. Control animals received saline vehicle (300 µL/kg, sc). A.) Body temperature data are change from baseline. Filled symbols indicate significant difference from the control group (*p* < 0.05, one-way ANOVA, Tukey’s HSD). * = significantly different from *para*-fluorofentanyl treatment group (*p* < 0.05). ‡ = significantly different from fentanyl treatment group (*p* < 0.05, one-way ANOVA, Tukey’s HSD post hoc), Each value is the mean ± SEM (*n* = 5–6). Average baseline temperature = 37.6 ± 0.1 ℃ (*n* = 17) B.) Maximal temperature change following sc. drug administration * = significantly different from fentanyl group (*p* = 0.017 with *t* = 2.873, df = 10). Each value is the mean ± SEM (*n* = 6)

Figure 4A shows the normalized change in core body temperature over time for all treatment groups. Both fentanyl and pFF (F_(2,14)_ = 50.47, p < 0.001) displayed hypothermic responses when compared to the control group at 30 min (one-way ANOVA, Tukey’s HSD post hoc). At 60 min, fentanyl and pFF displayed significant (F_(2,14)_ = 75.5, p < 0.001) decreases in body temperature when compared to the control group (one-way ANOVA, Tukey’s HSD post hoc). Also at the 60-min time point, the change in core body temperature for pFF was significantly lower than fentanyl (F_(2,14)_ = 75.5, p = 0.016). Fentanyl (F_(2,14)_ = 19.85, p = 0.003) and pFF (F_(2,14)_ = 19.85, p < 0.001) still displayed significant decreases in core body temperatures when compared to the control group (one-way ANOVA, Tukey’s HSD post hoc) at 120 min post-dosing. Both compounds displayed no significant differences in change in core body temperature when compared with the control group at 240 min or 480 min. Figure 4B displays the maximum change from baseline for both fentanyl and pFF. Using a two-tailed t test, pFF resulted in a significantly lower −5.6 ℃ (*p* = 0.017, t = 2.873, df = 10) maximum change in temperature when compared to fentanyl (−4.1 ℃).

### Brain region concentrations

**Fig. 5:**
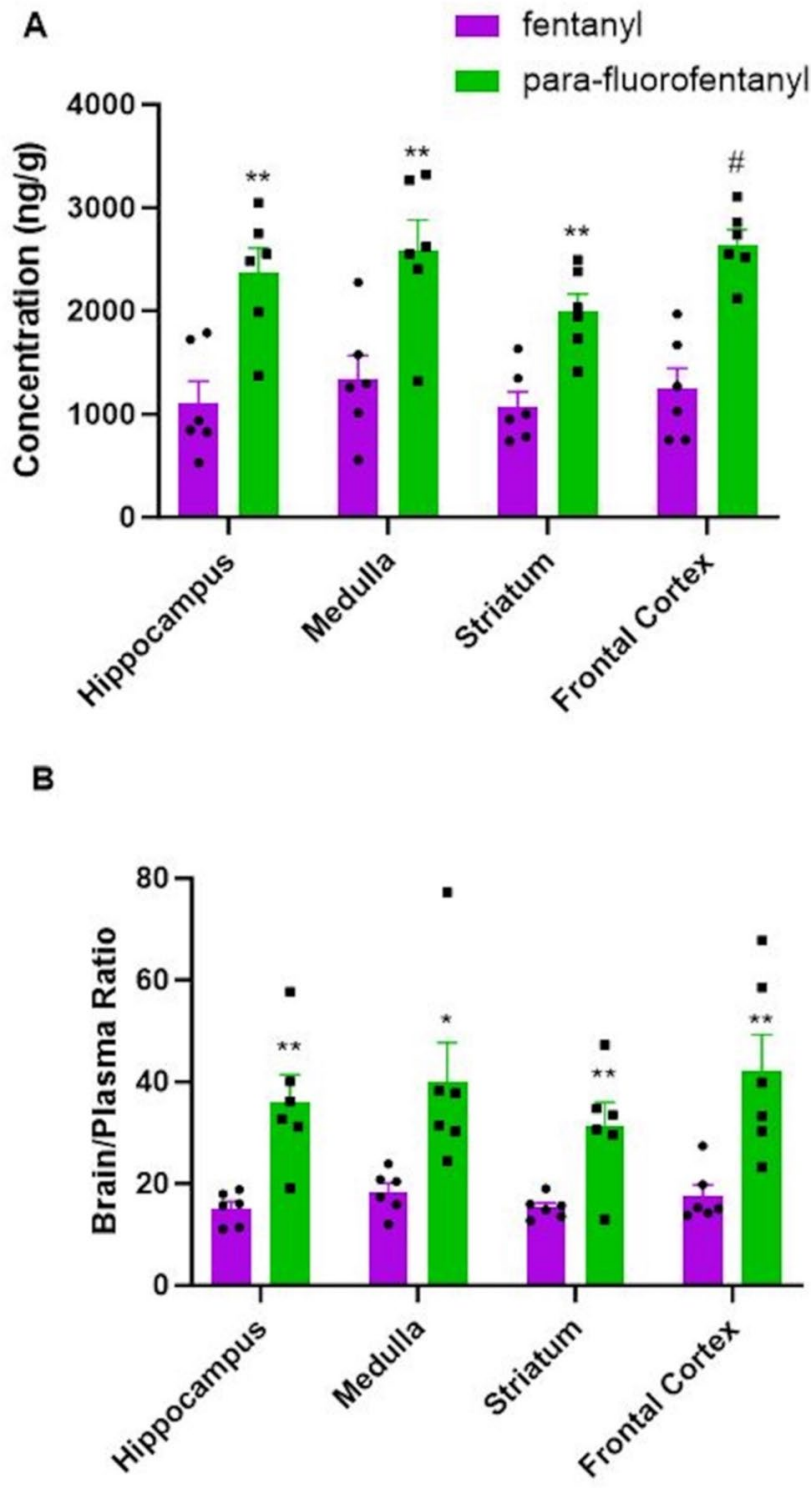
Brain region concentrations and brain to plasma ratios for fentanyl and *para*-fluorofentanyl. Both compounds were dosed at 300 µg/kg sc. A.) Hippocampus, medulla, striatum and frontal cortex concentrations for fentanyl and *para*-fluorofentanyl. ** = significantly different from corresponding fentanyl region (*p* < 0.01, Student’s *t* test). # = significantly different from corresponding fentanyl region (*p* < 0.001, Student’s t test). Each value is the mean ± SEM (*n* = 6). B.) Brain to plasma ratios for hippocampus, medulla, striatum and frontal cortex for fentanyl and *para*-fluorofentanyl. * = significantly different from corresponding fentanyl region (*p* < 0.05, Student’s *t* test). ** = significantly different from corresponding fentanyl region (*p* < 0.01, Student’s *t* test). Each value is the mean ± SEM (*n* = 6)

Figure 5A displays the brain concentrations of fentanyl and pFF for each brain region (hippocampus, medulla, striatum, frontal cortex) 1 h after treatment. All brain regions displayed significantly greater concentrations in the pFF treatment group when compared to the fentanyl treatment group (p = 0.003 for the hippocampus, p = 0.008 for the medulla, p = 0.002 for the striatum, p = 0.0002 for the frontal cortex, all compared using a Student’s t test). Figure 5B displays the brain to plasma ratios of each brain region for the fentanyl and pFF groups. The brain to plasma ratios displayed a similar trend as the brain region concentrations with the pFF ratios being significantly greater than the fentanyl ratios (p = 0.003 for the hippocampus, p = 0.022 for the medulla, p = 0.006 for the striatum, p = 0.008 for the frontal cortex, all compared using a Student’s t test).

## Discussion

In the present study, fentanyl and pFF were found to be equally active at the tested dose for analgesia as assessed by tail flick response, with both compounds displaying an analgesic response for 240 min post-dosing. Fentanyl and pFF both elicited a hypothermic response for up to 120 min post-treatment; however, pFF induced a greater hypothermia at 60 min and an augmented maximum change in temperature when compared to fentanyl. Although no PK differences or plasma concentration differences were observed between the two drugs, pFF displayed double the brain concentrations of fentanyl and greater brain to plasma ratios than fentanyl in each region. The plasma concentrations and PK parameters of fentanyl are in agreement with those of our previous study (Canfield and Sprague 2023). In addition to our previous PK findings, fentanyl and pFF also displayed similar PK parameters to those of cyclopropylfentanyl, another prominent IMF. Bergh et al. (2021) determined cyclopropylfentanyl to have a similar T_1/2_ of 94.5 min, compared to fentanyl in the present study (1.10 h) and pFF (1.12 h) In addition, cyclopropylfentanyl also displayed a similar C_max_ of 90.83 ng/mL to that of fentanyl (82.12 ng/mL) and pFF (112.57 ng/mL) as shown in our present findings (Bergh et al. 2021). The tail flick analgesic response and temperature findings were also in agreement with our previous study with fentanyl displaying analgesic effects up to 240 min in both studies and displaying hypothermic responses up to 120 min in both studies for the same 300 μg/kg dose (Canfield and Sprague 2023).

Varshneya et al. (2023) compared the PD effects of fentanyl and pFF in a mouse model, however, PK parameters and core body temperature were not examined. Those authors measured analgesia using a warm water bath and found pFF and fentanyl to be equipotent to one another with both compounds producing an approximate 50% MPE at a 0.1 mg/kg subcutaneous dose and an approximate 100% MPE effect at a 1 mg/kg subcutaneous dose. Similar to our current findings, Varshneya et al. (2023) also found fentanyl and pFF to be equally active at the tested doses throughout the duration of the study and to provide analgesic responses for up to 240 min. While not calculated in the present study, Varshneya et al. (2023) also determined pFF to have a potency ratio of 1.30 to fentanyl indicating approximate equipotency. This differs from the potency ratio as determined by Higashikawa and Suzuki (2008). Those authors found pFF to have a potency ratio of 0.29 as compared to fentanyl. Differences in methods could explain the potency difference between the two studies as Higashikawa and Suzuki used an oral route of administration for pFF paired with writhing episodes induced by 0.1 mL of acetic acid to assess potency instead of the subcutaneous dosing route and warm water tail withdrawal methods used by Varshneya et al. (Higashikawa and Suzuki 2008; Varshneya, et al. 2023). One limitation in making a direct comparison between our study and that of Varshneya et al., (2023) is that we used a single 300 μg/kg dose of fentanyl and pFF. However, this dose was based on previous IMF PK and PD studies. Bergh et al., (2021) examined the PK and PD of cyclopropylfentanyl at 30, 100 and 300 μg/kg. Subsequent to those findings, we used a 300 μg/kg dose to examine the effects of altering side change length on the PK and PD effects of acetylfentanyl, butyrylfentanyl, cyclopropylfentanyl, fentanyl and valerylfentanyl (Canfield & Sprague 2023). In that study, our findings with cyclopropylfentanyl regarding the PK and PD responses were consistent with those of Bergh et al. (2021). In addition, we utilized a rat model, while Varshneya et al. (2023) utilized a mouse model. Mice and rats have been shown to have differential responses to opioids (Uddin et al. 2021).

To date, we believe we have reported the effects of pFF on core body temperature for the first time. Thermoregulatory effects of opioids have been shown to be naloxone reversible and are, therefore, MOR receptor mediated as well as dose dependent, with lower doses eliciting a hyperthermic response and higher doses demonstrating a hypothermic response (Adler et al. 1988; Geller et al. 1983; Handler et al. 1992; Kiyatkin and Choi 2024). The mechanism for hypothermia is believed to also involve ∝_1_ receptor antagonism (Canfield and Sprague 2023). ∝_1_ antagonism has been shown to result in vasodilation and increased heat dissipation leading to a lower body temperature (Mills et al. 2004). Sohn et al. (2005) showed that fentanyl has structural portions in common with ∝_1_ antagonists and agonists and the hypothermic effects indicate that fentanyl can act as an ∝_1_ antagonist, in addition to the MOR agonist-mediated hypothermic effects seen with higher doses of opioids (Sohn et al. 2005). The addition of the fluorine functional group does not appear to alter these ∝_1_ antagonistic or MOR agonistic properties of fentanyl as the same amount and even greater hypothermia was seen with pFF. Wong et al. (2017) determined that the hypothermic effects of carfentanil parallel decreased respiration, decreased blood pressure and bradycardia in rats, and subsequently, Bergh et al. (2019) utilized hypothermia as a surrogate marker for opioid toxicity in rats (Bergh et al. 2019; Wong et al. 2017). Based upon these findings, we also used hypothermia as a surrogate marker for opioid toxicity and noted that these effects diminished sooner than the analgesic effects. pFF had a greater maximum temperature change as compared to fentanyl and a greater temperature decrease at 60 min as compared to fentanyl. Together these findings indicate that while equally active at the tested dose to fentanyl in terms of analgesia, pFF does have a greater hypothermic response and, therefore, a potential for greater toxicity than fentanyl.

In the present study, pFF displayed significantly greater brain concentrations and brain to plasma ratios as compared to fentanyl. The brain to plasma ratio indicates the total brain concentration of a drug compared to the blood concentration, with compounds that exhibit a higher brain to plasma ratio demonstrating the ability to cross the blood brain barrier and act on the central nervous system (CNS). For a specific compound, having a brain to plasma ratio close to or slightly greater than 1.0 indicates that the compound can freely cross the blood brain barrier, while a number that is much greater than 1.0 indicates that the compound could be accumulating in brain tissue (Kulkarni et al. 2015) We believe this is the first time brain specific regions have been assessed for fentanyl and pFF concentrations and of particular interest were the hippocampus, striatum and medulla. The hippocampus and striatum are key parts of the mesolimbic dopamine reward system with the hippocampus being involved in memory formation and the striatum containing the nucleus accumbens, a key center for reward via dopaminergic projections (Nestler 2001, 2005; Volkow et al. 2004). pFF displaying higher concentrations indicates that this fentanyl analog in particular permeates the BBB quicker than fentanyl and to a greater extent leading to the potential for increased MOR activation in these brain regions. Therefore, greater amounts of pFF in the hippocampus and striatum could result in an increased potential for abuse as these two brain regions play a role in reward processing, especially for opioids (Fields and Margolis, 2015). However, to date, this has not been studied. In addition, we examined the brain concentrations of both compounds in the medulla as this is a key brain region involved in respiration (Webster and Karan 2020). Fatal opioid overdoses typically result from a decrease in respiration and greater amounts of pFF in the medulla could lead to enhanced MOR activation resulting in an increased potential for respiratory depression and, therefore, a greater potential for fatal overdose (Baertsch et al. 2021; Pattinson 2008; Saunders et al. 2022). Interestingly, the increased hypothermic response and overall greater brain concentrations of pFF as compared to fentanyl does not appear to be due to differences in PK parameters as no differences were observed between the two compounds. The lack of difference in PK profile between the two and the significantly greater brain concentrations indicates that the increased potential for toxicity is likely due to increased permeability of the BBB and greater exposure in the brain.

Overall, the increased potential for toxicity as indicated by greater hypothermia and the increased concentrations of pFF in the brain indicate that pFF, a prominent fentanyl analog, may have a greater abuse potential and could be more dangerous than its predecessor. Given that 99% of pFF samples analyzed by crime laboratories also contain fentanyl (Canfield et al. 2024), there is a high likelihood for potentiation of overall toxicity.

## Supplementary Information

Below is the link to the electronic supplementary material.

Supplementary file1 (DOCX 304 KB)

## Data Availability

Data can be made available upon request.
