# Supplementary material for: In vivo pharmacokinetic, pharmacodynamic and brain concentration comparison of fentanyl and para-fluorofentanyl in rats
Source: Arch Toxicol. 2024 Oct 17;99(1):287–97. doi: 10.1007/s00204-024-03887-z (PMC11748481; doi:10.1007/s00204-024-03887-z)

**Supplementary Table 1: Retention Time (RT), precursor and product ions, Q1 pre bias voltages, collision energy voltages (CE), and Q3 pre bias voltages**

| **Compound** | **RT (min)** | **Precursor Ion (m/z)** | **Product Ions (m/z)** | **Q1 Pre Bias (V)** | **CE (V)** | **Q3 Pre Bias (V)** |
| --- | --- | --- | --- | --- | --- | --- |
| Fentanyl | 4.0 | 337.10 | 188.15* | -10.0 | -23.0 | -20.0 |
|  |  |  | 132.25 | -10.0 | -31.0 | -14.0 |
| *para*-fluorofentanyl | 3.9 | 355.20 | 188.05* | -28.0 | -24.0 | -21.0 |
|  |  |  | 105.10 | -28.0 | -37.0 | -22.0 |
| Fentanyl-D5 | 4.0 | 342.30 | 188.20 | -8.0 | -22.0 | -13.0 |

*****Indicates quantifying ion.

**Supplementary Table 2: *para*-fluorofentanyl plasma validation results**

| **R^2^ (range, n=5)** | | | | | | **LOD/LLOQ Bias (%, n=9)** | | | **LOD/LLOQ Within-run precision (%CV, n=3)** | | **LOD/LLOQ Between-run precision (%CV, n=9)** | |
| --- | --- | --- | --- | --- | --- | --- | --- | --- | --- | --- | --- | --- |
| 0.9921-0.9983 | | | | | | 2.2 | | | 3.8 | | 8.9 | |
| **Bias (%, n=15)** | | | **Within-run precision (%CV, n=3)** | | | **Between-run precision (%CV, n=15)** | | | **Matrix Effects (%, n=10)** | | **Recovery (%, n=10)** | |
| **LQC** | **MQC** | **HQC** | **LQC** | **MQC** | **HQC** | **LQC** | **MQC** | **HQC** | **LQC** | **HQC** | **LQC** | **HQC** |
| -9.1 | 7.2 | 4.2 | 4.2 | 4.7 | 5.4 | 5.4 | 4.9 | 4.8 | 19.4 | 18.5 | 94.8 | 87.2 |

**Supplementary Table 3: Fentanyl and *para*-fluorofentanyl brain matrix effects**

| **Compound** | **Matrix Effects (%, n=5)** |
| --- | --- |
| Fentanyl | 14.6 |
| *para-*fluorofentanyl | -2.9 |

**Supplementary Figure 1: Fentanyl plasma standard (A) and plasma sample (B) representative chromatograms and *para-*fluorofentanyl plasma standard (C) and plasma sample (D) representative chromatograms**

**A**


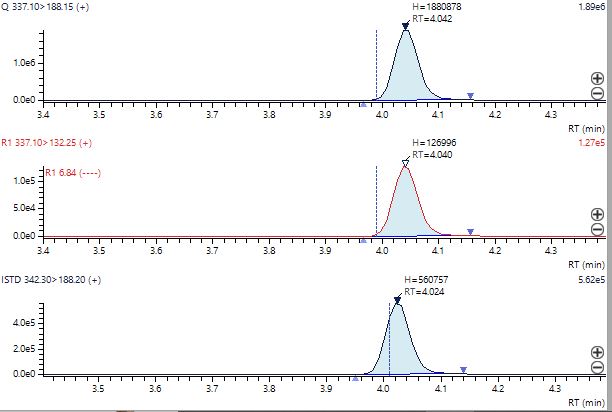


**B**


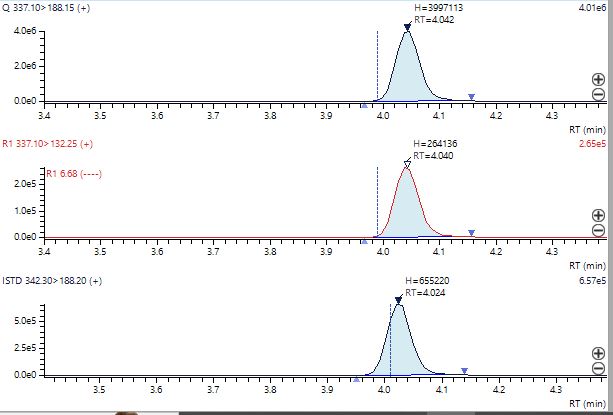


**C**


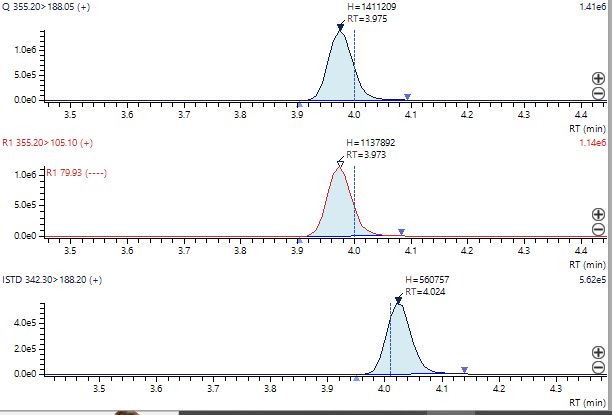


**D**


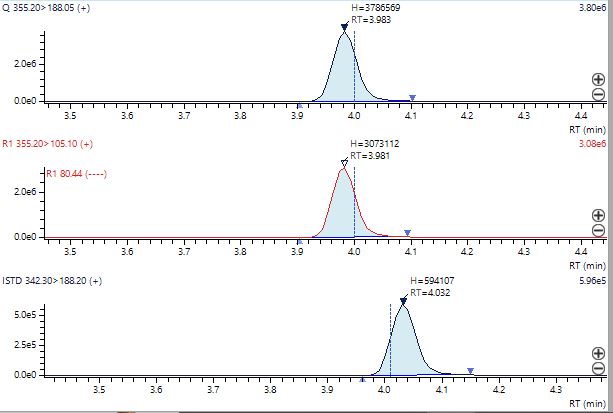


**Supplementary Figure 2: Fentanyl brain region standard (A) and brain region sample (B) representative chromatograms and *para-*fluorofentanyl brain region standard (C) and brain region sample (D) representative chromatograms**

**A**


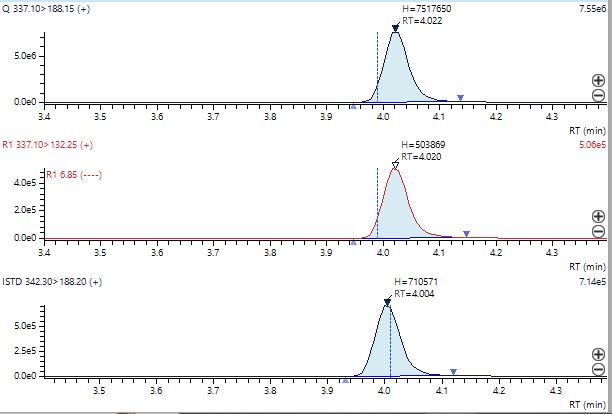


**B**


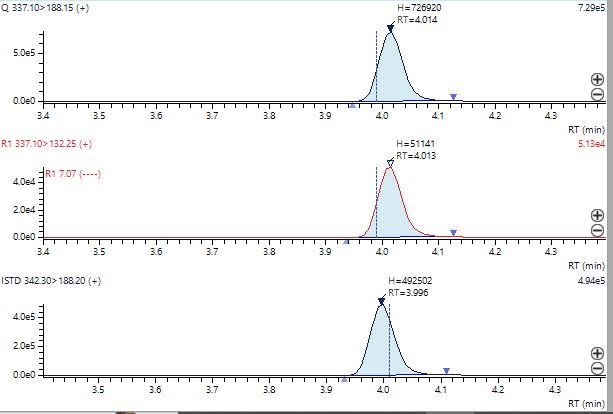


**C**


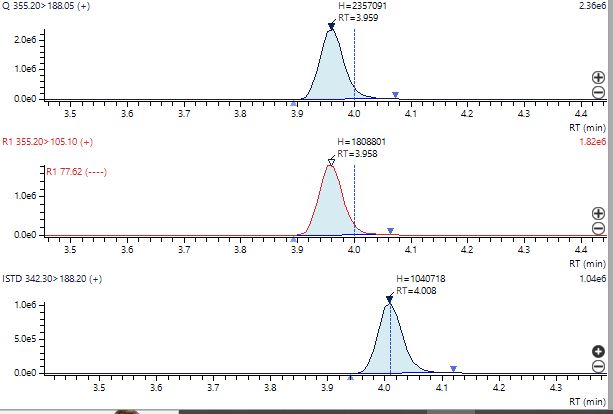


**D**


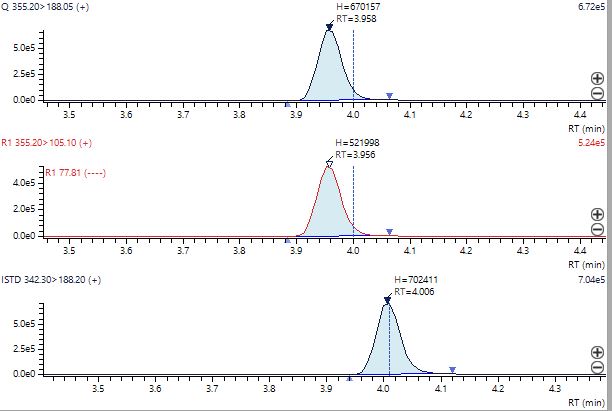

Supplement: Supplementary file 1 — Supplementary file1 (DOCX 304 KB) [file 204_2024_3887_MOESM1_ESM.docx]
